# Supplementation of *Bacillus subtilis* and *Lactobacillus casei* to increase growth performance and immune system of catfish (*Clarias gariepinus*) due to *Aeromonas hydrophila* infection

**DOI:** 10.14202/vetworld.2024.602-611

**Published:** 2024-03-17

**Authors:** Nurul Aini, Dini Sarafina Yulia Rosa Putri, Divany Hunaimatul Achhlam, Fatimah Fatimah, Sapto Andriyono, Dyah Hariani, Hoang Dang Khoa Do, Sri Puji Astuti Wahyuningsih

**Affiliations:** 1 Doctoral Mathematics and Natural Sciences Study Program, Faculty of Science and Technology, Universitas Airlangga, Surabaya, Indonesia; 2 Department of Agricultural Technology, KH University. A. Wahab Hasbullah, Jombang, Indonesia; 3 Department of Biology, Faculty of Science and Technology, Universitas Airlangga, Surabaya, Indonesia; 4 University Center of Excellence Research Center for Bio-Molecule Engineering, Universitas Airlangga, Surabaya, Indonesia; 5 Department of Marine, Faculty of Fisheries and Marine Sciences, Universitas Airlangga, Surabaya, Indonesia; 6 Department of Biology, Faculty of Mathematics and Natural Sciences, Surabaya State University, Surabaya, Indonesia; 7 NTT Hi-Tech Institute, Nguyen Tat Thanh University, Ho Chi Minh City, Vietnam

**Keywords:** catfish, fish stocks and fisheries management, growth parameters, immune response, probiotic

## Abstract

**Background and Aim:**

Catfish has a high economic value and is popular among consumers. To ensure well-stocked catfish stocks, good fisheries management must also be ensured. The high demand for catfish must be supplemented by preventive measures against pathogenic bacterial infections using probiotics with high potential for *Lactobacillus casei* and *Bacillus subtilis*. The aim of this study was to determine the effect of probiotic supplementation consisting of a combination of *L. casei* and *B. subtilis* probiotics on the growth, immune system, water quality, proximate value of feed, and body composition of catfish infected with *Aeromonas hydrophila*.

**Materials and Methods:**

This study used a completely randomized study with eight treatments and three replications. The manipulated factor was the probiotic concentration [0% (A), 0.5% (B), 10% (C), and 15% (D)] in groups of catfish infected and uninfected with *A. hydrophila*. Combination of *B. subtilis*, an*d L. casei* that were use*d* in a 1:1 ratio of 10^8^ colony forming unit/mL. The study lasted for 42 days. On the 35^th^ day, *A. hydrophila* was infected by intramuscular injection into fish. The Statistical Package for the Social Sciences (SPSS) software version 23.0 (IBM SPSS Statistics) was used to analyze data on growth, immune system, and water quality.

**Results:**

Providing probiotics in feed can increase the nutritional value of feed based on proximate test results. There were significant differences in average daily gain (ADG), feed conversion ratio (FCR), and survival rate (SR) parameters in the group of catfish infected with *A. hydrophila* (p > 0.05); however, there were no significant differences in final body weight, specific growth rate (SGR), and percentage weight gain. Interleukin-1β (IL-1β) levels were significantly different between treatments C and D. The tumor necrosis factor (TNF) α parameters were significantly different between treatments A and C, whereas the phagocytic activity of treatment A was significantly different from that of treatment D. There was a significant difference (p > 0.05) in the growth parameters of SGR, ADG, and FCR in the group of fish that were not infected with *A. hydrophila*, with the best treatment being a probiotic concentration of 15%, but there was no significant difference in the SR parameters. IL-1β and TNF-α levels significantly differed between E and E0 (15% probiotics) but were not significantly different in terms of phagocytosis parameters.

**Conclusion:**

Based on the results of this study, it can be concluded that using a combination of probiotics *L. casei* and *B. subtilis* can improve the growth, immune system, water quality, proximate value of feed, and body composition of catfish infected with *A. hydrophila*.

## Introduction

Aquaculture activities significantly contribute to fulfilling human and animal protein requirements [1]. Catfish is one of these aquaculture products [2]. However, various problems are always encountered in the process of catfish cultivation. For example, feed prices are increasing [3], environmental quality is decreasing [4], and the mass death of fish is due to pathogenic bacteria. *Aeromonas hydrophila* is a disease infection that often attacks catfish [5]. These pathogenic bacteria can cause serious diseases, such as Motile *Aeromonas* septicemia [6], catfish enteric septicemia, and columnaris disease [7].

The problem of infection in catfish can be solved using antibiotics [2]. However, the emergence of new strains of pathogenic microbes that are antimicrobial resistant could reduce the quality of fish meat and enable the presence of antibiotics in the consumer’s body resulting from the consumption of antibiotics-containing fish. [8–10]. Of course, it is not comparable to the long-term benefits of antibiotics. To overcome the excessive use of antibiotics, probiotics can be used [11, 12]. Studies related to the use of probiotics in humans and animals [13, 14] as well as in aquaculture, especially in catfish [15, 16] have been conducted.

Probiotics are good microorganisms that are safe for the host. The addition of probiotics (either live or dead) to an aquaculture system can have a beneficial impact [17]. The benefits of using probiotics for fish include improving the immune system [18], increasing fish growth and productivity [19, 20], improving water quality, reducing ammonia residues and bioremediation agents [21, 22], increasing feed efficiency [23], increasing resistance to infectious diseases [5, 24], improving the balance of microflora in the fish digestive tract [25, 26], and increasing the expression of genes related to immunity, growth, and nutrient metabolism [27].

*Lactobacillus casei* and *Bacillus subtilis* have been widely used in aquaculture [28]. According to previous studies by Kuebutornye [29] and Safari *et al*. [30], the addition of *L. casei* and *B. subtilis* probiotics separately has a good effect on fish. A combination of different types of probiotics with a suitable composition can have a better effect than a single type of probiotic [31, 32].

This study aimed to determine the effect of combining two types of probiotics, *L. casei* and *B. subtilis* on growth, feed efficiency, immune system, and proximate analysis of feed and meat infected with *A. hydrophila*.

## Materials and Methods

### Ethical approval

This study was approved by the Animal Care and Use Committee, Faculty of Veterinary Medicine, Airlangga University (No: 2.KEH.073.05.2023).

### Study period and location

This study was conducted from July 2023 to October 2023 in the Microbiology Laboratory of the Department of Biology, Faculty of Science and Technology, Airlangga University. Fish immune system parameters were measured in the Molecular Genetics Laboratory. Catfish were reared at the Kumis Lele Farm Panjang Jiwo, Surabaya, Indonesia.

### Probiotic and diet preparation

The probiotic bacteria *L. casei* and *B. subtilis* were recultured in MRSB media (De Man–Regosa–Sharpe Broth, HiMedia, USA), and *B. subtilis* and *A. hydrophila* were recultured in NB media (Nutrient Broth, NB, HiMedia, USA). Each culture was incubated for 48 h at 35°C. After incubation, the optical density value was observed on a spectrophotometer and cells were grown on MRSA media (De Man–Regosa–Sharpe Agar, HiMedia) for *L. casei* and NA media (Nutrient Agar, HiMedia) for *B. subtilis* and *A. hydrophila*) until a cell density of 10^8^ colony-forming units/mL was obtained. The total plate count method is used to determine the number of cell densities.

The commercial feed Prima Feed LP-1 SP (PT Matahari Sakti, Surabaya, Indonesia) was used in this study. The pellets were placed in a basin and sprayed with probiotics according to the treatment conditions. Probiotic doses of 0%, 5%, 10%, and 15% were administered. Add probiotics using a spray bottle and spray. The sprayed feed was then homogenized and left for 18–24 h in a closed container for the feed fermentation process [11]. Feed ready to be given to fish is subjected to a proximate analysis to determine its nutritional value.

Feed that has been added with probiotics will be tested for the attachment of probiotics to the feed by gradually diluting 1 g of each feed treatment. The dilution results were obtained using MRSA and NA media. Colonies that appear are counted using a colony counter. The number of probiotic bacteria attached to the feed after spraying and fermenting for 24 h was determined.

### Catfish management

Catfish were obtained from the Kumis Lele Farm, Panjang Jiwo Surabaya. The purchased fish are in good health and are well maintained in accordance with the maintenance procedures recommended by the Directorate General of Indonesian Aquaculture, Ministry of Fisheries and Maritime Affairs.

The aquarium used in this study has a capacity of 84 L, totaling 24 units. Previously, the aquarium was washed with fresh water and disinfected with chlorine at a concentration of 10 PPM (parts per million) to remove the attached bacteria and fungi. The aquarium is then washed with water until clean and dried in the sun for approximately 24 h. In the next stage, each aquarium was filled with 70 L of water and equipped with an aerator.

Catfish with a length of 19–23 cm and an average weight of 80–100 g were used. Before the acclimation process, the fish were soaked in methylene blue solution for 25 L of water at a dose of 1 drop of methylene blue. The next step was to place the fish in the aquarium for 7–24 h for acclimation. During acclimation, the fish were given PT Matahari Sakti Prima Feed LP-1 SP with 33% protein content. In the next stage, the test fish are randomly collected and placed into aquariums at a density of eight fish per aquarium.

Fish were kept in aquariums with a water volume of 70 L with an aerator aerating each aquarium. Each aquarium contains eight fish. The feeding rate was twice a day at 3% of the catfish’s body weight [33]. Fish were maintained for 42 days. The following are the research treatments:

- A: 0% probiotics and infection with pathogen
- B: 5% probiotics and infection with pathogen
- C: 10% probiotics and infection with pathogen
- D: 15% probiotics and infection with pathogen
- A0: 0% probiotics and uninfected with pathogen
- B0: 5% probiotics and uninfected with pathogen
- C0: 10% probiotics and uninfected with pathogen
- D0: 15% probiotics and uninfected with pathogen

### Challenge test

On the 35^th^ day of rearing, catfish were intraperitoneally infected with *A. hydrophila* at 0.1 mL 10^8^ colony forming unit/mL. Fish that have not been infected with pathogens will be injected with 0.1 mL of phosphate-buffered saline solution. After infection, the fish were kept for 7 days with daily intensive observation.

### Analysis of body composition of the catfish

At the end of the study, proximate value of 5 g catfish body was determined. Tests were conducted as described by Niu *et al*. [34] method at the Chemical Laboratory, Faculty of Fisheries and Maritime Affairs, Airlangga University.

### Observation of fish growth

The weight of the fish at the beginning and end of the study was measured to determine the growth performance of the fish. Growth parameters such as initial body weight (IBW), final body weight (FBW), percent weight gain (PWG), average daily gain (ADG), specific growth rate (SGR), feed conversion ratio (FCR), and survival rate (SR) were calculated using the following formula:

PWG (%) = 100 × (FBW - IBW)/IBW

SGR (%/days) = 100 × (ln FBW - ln IBW)/days

ADG = (FBW-IBW)/rearing period

FCR = Dry feed consumed/weight gain

SR (%) = 100 × (final fish number/initial fish number).

### Procedure for measuring the fish immune system

Blood samples were collected 7 days after the infection. Fish blood was collected using a syringe from the caudal vein at the base of the fish’s tail. Blood was placed in a sterile microtube. Blood was left at room temperature (30°C) for 2 h. Next, the blood serum was isolated by centrifugation at 1678× *g* RPM for 10 min until two phases were formed and the supernatant was collected. Measurement of interleukin (IL)-1β, tumor necrosis factor (TNF)-α, lysozyme enzyme levels were conducted using the fish IL-1β enzyme-linked immunosorbent assay (ELISA) kit (BT Lab, Zhejiang, China), the Fish TNF-α ELISA kit (BT Lab), and the ELISA Kit Fish lysozyme ELISA kit (BT Lab), respectively, according to the manufacturers’ instructions.

### Water quality measurement procedures

Measurements of water quality are carried out every 3 days. The test parameters and equipment used in this research are as follows: Temperature is measured using a Celsius scale thermometer, dissolved oxygen (DO) is calculated using a DO meter, pH is measured using a pH meter, and ammonia, nitrate, and nitrite levels are measured using MQuant Ammonium Test Merck KGaA, MColortest Nitrate Test Merck KGaA, and MQuant Nitrite Test Merck KGaA. Phosphate concentrations were measured using Merck MQuant Phosphate Test (Sigma-Aldrich, Germany).

### Statistical analysis

This study used a completely randomized design with experiments performed in triplicate. Data obtained from this study were subjected to analysis of variance using the Statistical Package for the Social Sciences software version 23.0 (IBM Corp., Armonk, NY, USA). The *post hoc* Duncan test was performed if a significant difference in the mean was identified (p < 0.05).

## Results

### Proximate analysis of the feed

**Figure-1:**
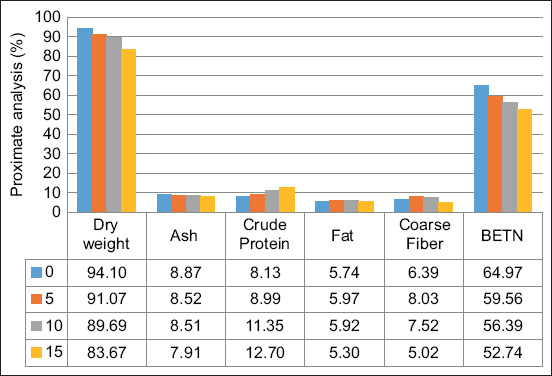
Proximate analysis value of feed after being treated with probiotics with various concentrations of 0%, 5%, 10%, and 15% of probiotics.

As shown in Figure-1, the addition of probiotics to feed influences the proximate analysis of feed. The addition of 0% probiotics resulted in the lowest crude protein content and the highest crude fiber content compared with the other treatments. In addition, feed with a higher concentration of probiotics produces feed with a higher crude protein content and lower crude fiber content. The addition of 15% probiotics resulted in the feed having the best proximate value compared to the other treatments, namely protein (12.7%), crude fat (5.3%), crude fiber (5.02%), and extract material without nitrogen (52.74%) values.

### Growth performance

Based on these observations, it can be seen that the FBW of the fish during the 42^nd^ day of rearing had a high increase. The growth parameters of fish after probiotic treatment are listed in Tables-1 and 2.

**Table-1:** Growth parameters in groups of fish infected with the pathogen *Aeromonas hydrophila*.

Treatments	IBW (g)	FBW (g)	PWG (%)	SGR (%/day)	ADG (g/fish/day)	FCR	SR (%)
A	79.38 ± 0.63	116.70 ± 3.64^a^	47.01 ± 3.50^ab^	0.92 ± 0.06^a^	0.89 ± 0.07^ab^	1.54 ± 0.09^b^	95.83 ± 7.22^ab^
B	78.33 ± 1.91	123.58 ± 0.84^a^	57.83 ± 4.31^a^	1.09 ± 0.07^bc^	1.08 ± 0.06^b^	1.48 ± 0.07^ab^	91.67 ± 7.22^ab^
C	83.38 ± 1.00	117.90 ± 1.90^a^	41.41 ± 0.72^a^	0.82 ± 0.01^a^	0.82 ± 0.02^ab^	1.63 ± 0.03^b^	87.50 ± 0.00^b^
D	81.33 ± 1.08	121.28 ± 0.98^a^	49.14 ± 3.06^a^	0.95 ± 0.05^a^	0.95 ± 0.05^a^	1.74 ± 0.01^c^	79.17 ± 7.22^a^

*Different letters indicate significant difference between treatment groups, IBW=Initial body weight, FBW=Final body weight, PWG=Percent weight gain, ADG=Average daily gain, SR=Survival rate

**Table-2:** Growth parameters in groups of fish not infected with the *Aeromonas hydrophila* pathogen.

Treatments	IBW (g)	FBW (g)	PWG (%)	SGR (%/day)	ADG (g/fish/day)	FCR	SR (%)
A0	81.67 ± 1.30	119.08 ± 3.78^a^	45.79 ± 2.36^ab^	0.90 ± 0.04^ab^	0.89 ± 0.06^ab^	1.51 ± 0.03^b^	100.00 ± 0.00^a^
B0	79.29 ± 0.51	119.38 ± 3.71^a^	50.54 ± 3.77^ab^	0.90 ± 0.36^ab^	0.95 ± 0.08^ab^	1.52 ± 0.03^b^	91.67 ± 7.22^ab^
C0	79.79 ± 1.30	115.81 ± 5.31^a^	45.16 ± 6.79^a^	0.74 ± 0.04^ab^	0.86 ± 0.13^ab^	1.50 ± 0.06^b^	95.83 ± 7.22^a^
D0	84.38 ± 2.25	140.57 ± 5.32^b^	66.79 ± 10.80^a^	1.11 ± 0.03^c^	1.34 ± 0.18^c^	1.40 ± 0.02^a^	95.83 ± 7.22^ab^

*Different letters indicate significant difference between treatment groups, IBW=Initial body weight, FBW=Final body weight, PWG=Percent weight gain, ADG=Average daily gain, SR=Survival rate

The analysis results showed no significant differences in almost all treatments with the addition of probiotics at various concentrations, except for treatment D with 15% probiotic. The FBW of the fish was higher than that of the fish infected by the pathogen.

Another growth parameter (PWG) showed that the 5% concentration of probiotics in the group of fish infected with pathogens was better than that in the other treatments, but this difference was not statistically significant. This pattern has also been observed in the uninfected fish groups.

In terms of SGR parameters, treatment B with the addition of 5% to the infected fish group was the best treatment (1.09 ± 0.07), but the best FCR value (1.34 ± 0.18) was observed. Meanwhile, for the group of fish that were not infected, the best treatment was the addition of 15% probiotics, i.e., SGR of 1.11 ± 0.03% and ADG of 1.34 ± 0.18, respectively. In addition, the SR parameter did not show a statistically significant difference between the treatments. However, the overall SR of the uninfected fish group was better than that of the infected fish group.

### Immune response parameters

The administration of *L. casei* and *B. subtilis* probiotics to fish feed influenced the levels of the cytokines IL-1β, TNF-α, lysozyme enzyme, and phagocytic activity in both infected and uninfected fish groups, as shown in Tables-3 and 4. The state of fish’s immune system influences SR in catfish A 15% probiotic concentration administered to catfish exposed to *A. hydrophila* (D) resulted in low levels of proinflammatory cytokines. These values were 37.85 ± 3.60 ng/mL and 5.37 ± 2.02 μg/mL, respectively. However, there was no significant difference between the two treatments from the other treatments. In addition, immune parameters such as lysozyme level and phagocytic activity were high. They differed significantly from control group A (without being given probiotics and being infected with pathogens) by 114.55 ± 11.91 ng/mL and 38.47 ± 3.84%, respectively.

**Table-3:** Immune system parameters of groups of fish infected with *Aeromonas hydrophila*.

Treatments	IL-1 (ng/mL)	TNF-α (μg/mL)	Lysozyme (ng/mL)	Phagocytic activity (%)
A	47.31 ± 8.47^ab^	7.04 ± 0.30^b^	82.89 ± 12.57^a^	21.38 ± 6.30^ab^
B	42.97 ± 7.84^ab^	3.50 ± 0.59^a^	106.88 ± 5.25^ab^	27.02 ± 3.44^bc^
C	53.03 ± 10.74^b^	5.56 ± 1.16^ab^	104.18 ± 11.20^b^	32.93 ± 4.31^cd^
D	37.85 ± 3.60^a^	5.37 ± 2.02^ab^	114.55 ± 11.91^b^	38.47 ± 3.84^d^

*Different letters indicate significant difference between treatment groups, IL=Interleukin, TNF=Tumor necrosis factor

**Table-4:** Immune system parameters of groups of fish not infected with *Aeromonas hydrophila*.

Treatments	IL-1 (ng/mL)	TNF-α (μg/mL)	Lysozyme (ng/mL)	Phagocytic activity (%)
A0	43.67 ± 3.14^a^	4.73 ± 0.93^a^	102.98 ± 8.33^bb^	16.57 ± 1.13^a^
B0	75.92 ± 1.22^c^	4.09 ± 0.50^b^	107.35 ± 6.96^b^	19.82 ± 2.13^a^
C0	78.32 ± 9.89^c^	4.00 ± 0.21^b^	116.87 ± 7.36^b^	21.53 ± 1.51^ab^
D0	72.40 ± 10.05^c^	6.96 ± 2.40^b^	144.95 ± 6.49^c^	22.98 ± 3.59^ab^

*Different letters indicate significant difference between treatment groups, IL=Interleukin, TNF=Tumor necrosis factor

In the second group, 5% probiotics were administered to fish that were not infected (A0), which reduced the IL-1β values by 43.67 ± 3.14 ng/mL. Treatment of non-infected fish (C0) with 10% probiotics resulted in lower levels of TNF-α compared to other treatments. Lysozyme enzyme levels and phagocytic activity increased with increasing probiotic concentrations (144.95 ± 6.49 ng/mL and 22.98 ± 3.59%, respectively).

### Body composition analysis

**Figure-2:**
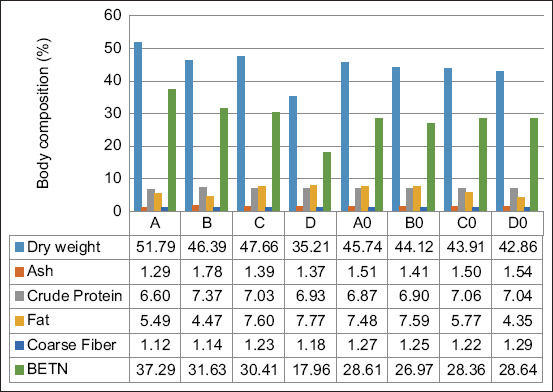
Results of analysis of catfish body composition.

At the end of catfish rearing, the fish are dissected and the flesh is used for proximate meat analysis. As shown in Figure-2, there was a trend of decreasing dry weight in the treatment group of fish infected with pathogens. The addition of 5% probiotics resulted in differences in crude protein levels between infected and uninfected fish. Crude protein levels tended to be higher in non-infected groups.

### Water quality

Tables-5 and 6 show the results of water quality measurements during the catfish rearing process with the parameters temperature, pH, and DO. All catfish ponds have temperature and pH within normal maintenance limits based on the SNI (Indonesian National Standard) catfish rearing rules [33]. The temperature range for keeping catfish is 25.1–28.9°C. The pH range is 7.5–8.6, and the DO value is between 7.5 and 9.3 mg/L.

**Table-5:** Water quality measurement results for temperature, pH, and DO.

Treatments	Temperature (°C)	pH	DO (mg/L)
A	26.1–28.9	7.5–8.6	8.1–9.3
B	25.7–28.3	7.5–8.6	7.5–8.6
C	25.7–28.9	7.5–8.5	8.0–8.9
D	25.7–28.3	7.5–8.6	8.1–8.9
A0	25.4–28.5	7.5–8.5	8.1–9.0
B0	25.1–28.1	7.5–8.6	7.9–8.3
C0	25.5–28.4	7.5–8.6	7.5–8.0
D0	26.1–28.9	7.5–8.6	8.1–9.3

DO=Dissolved oxygen

**Table-6:** Water quality measurement results for ammonia, nitrite, nitrate, and phosphate levels.

Treatments	Ammonia (mg/L)	Nitrite (mg/L)	Nitrate (cg/L)	Phosphate (mg/L)
A	1.00 ± 0.00^a^	0.18 ± 0.06^ab^	0.83 ± 0.14^ab^	1.17 ± 0.29^a^
B	1.00 ± 0.00^a^	0.18 ± 0.06^ab^	0.75 ± 0.00^ab^	1.17 ± 0.29^a^
C	0.83 ± 0.29^a^	0.15 ± 0.00^a^	0.67 ± 0.14^ab^	1.00 ± 0.00^a^
D	0.83 ± 0.29^a^	0.15 ± 0.00^a^	0.67 ± 0.14^ab^	1.00 ± 0.00^a^
A0	0.83 ± 0.29^a^	0.25 ± 0.00^ab^	0.83 ± 0.14^ab^	1.33 ± 0.29^a^
B0	1.00 ± 0.00^a^	0.25 ± 0.00^ab^	0.92 ± 0.14^b^	1.17 ± 0.29^a^
C0	0.83 ± 0.29^a^	0.22 ± 0.06^ab^	0.58 ± 0.14^a^	1.00 ± 0.00^a^
D0	1.00 ± 0.00^a^	0.15 ± 0.00^a^	0.58 ± 0.14^a^	1.00 ± 0.00^a^

*Different letters indicate significant difference between treatment groups

Table-6 shows other water quality parameters in the form of ammonia, nitrite, nitrate, and phosphate levels. The addition of different concentrations of probiotics to the feed does not appear to change water quality significantly. The test values for nitrite, nitrate, and phosphate showed a decreasing trend as the concentration of probiotics increased; however, no significant differences were observed in the treatment. Water quality is still included in the standards for safe catfish rearing during the fish rearing process, according to the Indonesian National Standard on catfish rearing [33].

## Discussion

Groups of *Lactobacillus* and *Bacillus* bacteria have often been used as ingredients for the production of probiotics in aquaculture. These two genera have been proven to have beneficial effects such as increasing skin mucus production, lysozyme activity, alkaline phosphatase activity, and protease activity [35], increasing the secretion of digestive enzymes [36], increasing growth, water quality [37], and improving the immune and digestive systems [38]. In addition, the use of probiotics can increase the protein content of fish feed [39].

In this study, the use of *L. casei and B. subtilis* bacteria increased the crude protein content. The higher the probiotic concentration, the higher the crude protein content. Fiber and BETN (extract material without nitrogen) levels were decreased due to the role of probiotics, which break down crude fiber in complex feed into simpler forms of carbohydrates. *L. casei* can produce lactases, proteases, peptidases, fructanases, amylases, bile salt hydrolases, phytases, and esterases [40]. In addition, *B. subtilis* can break down complex proteins, carbohydrates, and lipids from any source, allowing it to grow on almost any carbon and nitrogen source [41]. A combination of these two probiotics can optimize the degradation of nutrients in the feed. Luo *et al*. [1] reported that the combination of *B. subtilis* and *Lactobacillus* *plantarum* can increase the digestibility of nutrients in feed. Probiotics comprising a combination of *L. casei* YYL3 and *L. plantarum* YYL5 can also increase the growth of *Edwardsiella ictaluri*-infected catfish [41].

The immune system plays an important role for aquatic living creatures because there are pathogens that are ready to attack if the fish’s body defenses are weak [42]. Lysozyme, an immune system component, is essential for catfish to fight infection with the pathogen *A. hydrophila* [43]. Lysozyme can kill Gram-positive and Gram-negative bacteria through lytic activity and activation of complement and phagocytosis systems. Lysozyme kills bacterial cells by hydrolyzing N-acetylmuramate and N-acetylglucosamine acid, which are peptidoglycan layer components in the bacterial cell wall [44]. The addition of *L. casei* and *B. subtilis* probiotics increased the amount of lysozyme enzymes in catfish at a concentration of 15% in both infected and uninfected fish groups. According to research conducted by Rahman *et al*. [45], the addition of *Bacillus* sp. can increase lysozyme activity. Meidong *et al*. [46] reported that the use of *Bacillus aerius* B81e and *Lactobacillus paraplantarum* L34b-2 isolated from *Clarias gariepinus* can increase lysozyme levels in *Pangasius bocourti* fish that are kept for 60 days.

The addition of *L. casei* and *B. subtilis* probiotics can improve the immune system of catfish infected with *A. hydrophila*. Probiotic 15% is the best treatment that can increase phagocytic activity in the infected fish group after infection another researcher used *Bacillus* sp. to improve the immune system of catfish infected with *A. hydrophila* [47]. In addition, probiotics can reduce proinflammatory cytokines such as TNF-α and IL-1β. Previous studies have shown that different combinations of probiotics can increase phagocytic activity. For example, combined dietary probiotics *Lactococcus lactis* BFE920 and *L. plantarum* improved phagocytic activity in *Paralichthys olivaceus* [48]. A combination of probiotics can also increase phagocytic activity in *Lates calcarifer* infected with *A. hydrophila* [49].

Probiotics can increase fish growth [50, 51]. The probiotics enriched the nutrients in feed through fermentation, which plays a vital role in increasing the growth of treated fish [52]. On the basis of the research results, the higher the concentration of *L. casei* and *B. subtilis* probiotics applied, the better the impact of the feed on fish growth was found. The addition of 15% probiotics was the best treatment for reducing the FCR value in both *A. hydrophila*-infected and uninfected groups. Probiotics that enter the fish’s digestive tract help the macromolecular degradation process in feed through enzymes released by probiotics as well as by fish [53]. The results of the present study showed that this 15% concentration could cause an increase in SGR, ADG, and PWG values; however, it was not statistically different from the 5% probiotic treatment group. According to Amenyogbe *et al*. [54], feeding probiotics through the feed can increase growth, proximate analysis, and digestive enzyme activity in juvenile cobia (*Rachycentron canadum*) fish.

Catfish meat supplemented with the probiotics *L. casei* and *B. subtilis* exhibited different compositions, although not significantly different. There may be an increase in protein content due to increased deposition of nutrients. This is in accordance with Opiyo *et al*. [52], who reported increased protein content and decreased crude lipid content in *Oreochromis niloticus* and *Oncorhynchus mykiss* treated with additional probiotics. The addition of probiotic *Bacillus* spp. can improve the body composition value of catfish [55]. The higher protein content observed in this study could be attributed to more protein secreted by probiotics in the catfish intestines and the effective conversion of digested food into structural protein to build more muscle [52]. On the other hand, other studies have demonstrated that probiotic treatment has no significant effect on protein, lipid, or ash content [56]. Crude lipids were lower in fish supplemented with probiotics than in controls. This means that fish have more protein and less fat, which is desirable in aquaculture [9, 28]. The amount of probiotics used in the study indicated that only a small amount of probiotics was needed for improvement.

*L. casei* plays a role in improving water quality and increasing DO levels. DO levels are the most important parameters that support fish life in water [57]. As a probiotic, *B. subtilis* plays an important role in the nitrogen cycle through various processes such as ammonification, nitrification, and denitrification [58]. *B. subtilis* can reduce total ammonia nitrogen and NH_3_ values and can remove various types of nitrogen from cultivation wastewater [59]. In this study, the use of probiotics did not show significantly different results in improving water quality. However, *B. subtilis* and *Bacillus licheniformis* can improve water quality in shrimp infected with *Fusarium solani* [60–62]. A combination of *Bacillus* spp. and *Lactobacillus* spp. can improve water quality in hybrid grouper fish (*Epinephelus fuscoguttatus* and *Epinephelus polyphekadion*) by direct administration to water [37].

## Conclusion

A combination of *L. casei* and *B. subtilis* exhibited sufficient effects on the growth, immune system, water quality, feed, and body composition of catfish infected with *A. hydrophila*. These results provide fundamental data for further studies examining the effects of the combination of different bacteria on probiotic development.

## Authors’ Contributions

NA, SPAW, FF: Conception and design of the study and drafted and revised the manuscript. SA, DH, and HDKD: Analysis and interpretation of the data. NA, DSYRP, and DHA: Collected the data. SA and SPAW: Drafted and revised the manuscript. All authors have read, reviewed, and approved the final manuscript.
